# IFIT3 GENE EXPRESSION AND FUNCTION CONTRIBUTE TO HAND PATHOLOGY EVEN WITH ANTIRETROVIRAL THERAPIES (CARTS)

**DOI:** 10.1093/geroni/igac059.3024

**Published:** 2022-12-20

**Authors:** Ranjit Kumar Das, Deepa Roy, Nirakar Sahoo, Upal Roy

**Affiliations:** The University of Texas Rio Grande Valley, Edinburg, Texas, United States; The University of Texas Rio Grande Valley, Brownsville, Texas, United States; The University of Texas Rio Grande Valley, Edinburg, Texas, United States; The University of Texas Rio Grande Valley, Brownsville, Texas, United States

## Abstract

The advent of cART has revolutionized the management of HIV-1 infection and saved the lives of millions of people worldwide. Yet, HIV-Associated Neurocognitive Disorder (HAND) continues to be clinically relevant with aging people with HIV-1 (PWH). The underlying mechanism of HAND remains poorly understood. In this regard Signal transducer and activator of transcription 1(STAT1) and interferon-induced protein with tetratricopeptide repeats 3 (IFIT3) genes have been shown to induce neuroinflammation and neuronal cell death. However, the role of IFIT3 in HAND pathology has not been well established. Therefore, the present work investigated the significant role of IFIT3 gene in the development of HAND. In the current study, we used an in-vitro model to expose the neuroblastoma cell-line SH-SY5Y with clinically relevant cARTs drug and HIV Tat protein to observe the STAT1 and IFIT3 genes dysregulation. Furthermore, study also investigates the STAT1 and IFIT3 protein dysregulation through immunocytochemistry and western blot assay. Overall observation indicated that HIV-Tat protein upregulated gene expression whereas with the cART exposure there was a downregulation of STAT1 and IFIT3 genes. We next aim to investigate the role of STAT1 and IFIT3 genes in HIV-induced neuroinflammation. The current study has established IFIT3 as a biomarker marker for the detection of HAND.

